# Analytical Methods for Nanomaterial Determination in Biological Matrices

**DOI:** 10.3390/mps5040061

**Published:** 2022-07-15

**Authors:** Magdalini Vladitsi, Charalampia Nikolaou, Natasa P. Kalogiouri, Victoria F. Samanidou

**Affiliations:** Laboratory of Analytical Chemistry, Department of Chemistry, Aristotle University of Thessaloniki, GR-54124 Thessaloniki, Greece; vladitsi@chem.auth.gr (M.V.); cnikolaou@chem.auth.gr (C.N.); kalogiourin@chem.auth.gr (N.P.K.)

**Keywords:** nanoparticles, analytical chemistry, ICP-MS (inductively coupled plasma-mass spectrometry), AAS (Atomic Absorption Spectrometry), nanotechnology, instrumental analysis

## Abstract

Nanomaterials are materials in which at least one of the three dimensions ranges from 1 to 100 nm, according to the International Organization for Standardization (ISO). Nanomaterials can be categorized according to various parameters, such as their source, their shape, and their origin. Their increasing use in industrial settings, everyday items, electronic devices, etc. poses an environmental and biological risk that needs to be assessed and appropriately addressed. The development of reliable analytical methods for both characterization and quantification of nanomaterials in various matrices is essential. This review summarized the recent trends in analytical methodologies for the characterization and determination of nanoparticles in biological matrices.

## 1. Introduction

### 1.1. Nanomaterials

Materials in which at least one of the three dimensions ranges from 1 to 100 nm are characterized as nanomaterials, according to the International Organization for Standardization (ISO). Nanotechnology has been used since 2600 BC and includes several applications from the color of fabrics and window panels to the usage of nanowires by Middle Eastern metalsmiths. Richard Feynman was the first scientist to raise attention on nanotechnologies in 1959. However, it was not used until 1974 by Norio Taniguchi for semiconductor processes [1]. After the discovery of nanoscale materials and structures, the worldwide market enabled their usage and their profits led to the amount of USD 64.2 billion by 2019 [1].

Nanomaterials can be categorized according to various parameters. These parameters apply to their source, their dimensions, their materials of origin, or their possible toxicity level [2,3,4]. As for their origin, the categories that apply to their origin are anthropogenic or natural. The anthropogenic category is divided, in accordance with their intentional or unintentional formation, to incidental and engineered nanomaterials [1]. Those that have natural or incidental origin are named ultrafine particles and originated from erupting volcanos, breaking sea waves, sandstorms, forest fires, or soils [1]. Another origin of natural nanomaterials is a living organism, such as ferritin or the nanocrystalline constituent of bones. All the above are associated with magnetoreception and are considered to have a ferromagnetic crystalline structure [3,4,5].

Human activity is responsible for the production of unintentional products that include nanomaterials. Perfect examples of the sources of these products are internal combustion engines, incinerators, metal fume, polymer fume, food transformation processes, and electric motors [1,5]. These procedures include high temperature rapid cooling and the presence of vaporizable materials, which leads to the emission of incidental nanomaterials [1,2]. The difference between natural and incidental nanomaterials and manufactured nanomaterials is their controlled shape, dimension, and composition of the latter category. The manufactured nanomaterials can have multiple origins, for example, metal oxides, metals carbon, polymers, or semiconductors [1,4]. There is a variety in their design, and it depends on their desired functionality. Their surface can be treated or coated, and their form has a large variety including spheres, fibers, needles, wires, tubes, rods, rings, plates, shells, etc. [1,2,3,4,5,6,7]. Nanoparticles can be synthesized in situ or ex situ as has been reviewed by Guo et al. [8].

Metal nanoparticles are produced in the same size domain as proteins (1 to 100 nm) and have wide applications in the biomedical field. They can be used alone or in combination with other nanostructures to enhance signal amplification, increase sensitivity, and improve detection, and quantify different biomolecules [6,7,8,9]. Carbon-based nanomaterials are characterized by unique properties, such as mechanical strength, high conductivity, chemical versatility, and optical properties. Among carbon-based nanomaterials, carbon nanotubes and graphene are commonly used in chemical analysis, as has already been reviewed by Crevillen et al. [10].

The dimensionality of nanomaterials is used to classify them in a second way. When all the exterior dimension of the nanomaterial is less than 100 nm, it is considered zero-dimensional nanoparticle (0D). Quantum dots fall into this category as they are 10 nm diameter semiconductor nanocrystals that operate as a potential well and are used to confine electrons and holes in electronics [1,2,3,4,5]. Full spheres, dendrimers (which are highly symmetrical), branching macromolecules, and hollow spheres are all included as the 0D nanomaterials. Composed of carbon (fullerenes), sodium tungsten, or gold; for example, cubes palladium; zinc oxide rings; vanadium oxide star-shaped crystal formations; and a variety of more complex floral or tree-like formations, such as those created with molybdenum disulfides, silicon carbide, or magnesium oxide, 0D nanomaterials are nanoparticles that have no dimensions. Individually, they can be utilized as a cell marker, in solution as an emulsifier, or as a reinforcing filler within a solid matrix [1].

At the nanoscale, one-dimensional (1D) nanomaterials have two exterior dimensions, with the third usually occurring at the microscale. Nanofibers, nanotubes, nanowires, and nanorods are examples of this. Inorganic materials, for example, have been used to make nanofibers. Aluminum oxide, titanium dioxide, carbon, titanium dioxide, or other polymers, such as nylon and polyurethane are perfect examples of 1D nanomaterials [3,4].

Nanotubes have a unique cylindrical crystalline shape with pentagons, hexagons, or heptagons as atoms. Carbon nanotubes are the most well-known, but boron nitride is used to make nanotubes. Sulfides of molybdenum, tungsten, or copper, as well as halides such as nickel chloride, Cadmium chloride, and cadmium iodine, are two types of cadmium. Nanowires have the highest ratio of any other material. 1D nanomaterials with a 1000:1, length-to-width ratio permits them to confine electrons in a lateral manner, which is useful in electronics.

In addition, thin films, nanocoatings, and nanoplates are 2D nanomaterials, which have only one exterior dimension at the nanoscale. Thin films have ceramic or metal coatings that are only a few atomic layers thick [10,11]. They are mostly employed in the fields of electrical engineering. In order to make electronic components, having insulating or conducting surfaces is necessary. Surfaces’ optical reflectivity or characteristics can be changed. Finally, 3D nanomaterials, such as nanocomposites and nanostructured nanomaterials, have internal nanoscale characteristics but no outward dimension at the nanoscale. Nanocomposites are multiphase solid materials containing at least one nanoscale exterior dimension in at least one phase [1,3,12,13,14]. Nanofillers spread in a bulk matrix are commonly referred to as nanocomposites. Nanofillers are available in 0D, 1D, and 2D nanomaterials. Polymers, ceramics, and metals can all be used as matrices. The final material can be a fiber, a film, or a volume in 1D, 2D, or 3D.

### 1.2. Chemical Composition

Nanomaterials can also be classified based on the chemical composition of their constituents [1]. They can be silver, copper, gold, or iron originated. Their applications are related to medical diagnostic, antibacterial agents, manufacturing, electronics, or conductive field. A second chemical form of nanomaterial is metal oxides. Perfect examples of this category are titanium dioxide and zinc oxide, which can be used in the cosmetics industry and as antibacterial or antistatic agents 

Another order of nanomaterials includes silicates, carbonates, and nitrides [1,4,7]. Carbon nano-objects represent one of the best-given orders of nanomaterials. They include graphene, carbon nanotubes, carbon nanofibers, and fullerenes. They can be used as electrodes for solar cells and organic LEDs while underpinning polymer matrix mixes. Polymers are the last order of nanomaterials [1,5]. Some types, including thermoplastics, thermosets, and elastomers, can be used to make nano-objects including nanospheres, nanofibers, and nanoporous membranes. Their operations are related to their structural compound, filtration membranes, membranes for energy cells, fire-resistant and antibacterial fabrics, optic factors, and flexible electrical rudiments [1,3,4]. Eventually, cellulose is a natural pseudopolymer composed of nanofibrils or nanocrystals, that have low cost, low viscosity, and veritably high mechanical resistance. They also have ease of functionalization, electrical conduction, and biodegradability and are related to mechanical, thermal, and flexible applications.

## 2. Occurrence of Nanomaterials in Biological Samples

The increasing use of nanomaterials in industrial settings, everyday items, electronic devices, etc., poses an environmental and biological risk that needs to be assessed and appropriately addressed. For this, we need reliable analytical methods for both characterization and quantification of nanomaterials in various matrices. Since there has been a growing interest in biological samples in recent years and the effect that nanoparticles have upon biological systems, we have put our focus on nanoparticle (NP) determination in biological matrices in this review. Analysis of biological matrices can be challenging as background noise is often high and analyte concentration can be quite low, which means the extraction of analytes is often necessary. That is the case for NPs as well. The extraction of NPs from their biological matrices is also a difficult case. Common pretreatment techniques are liquid-liquid extraction, centrifugation, dielectrophoresis, and field-flow fractionation [2].

### Nanoparticles in Biological Systems

In recent years, an increased interest has been shown in the nature of nanomaterial and biological systems interactions, also called ‘nano-bio’ interactions [3]. The systematic study of these interactions can help expand our knowledge and predict biological responses, thus influencing the design and usage of nanomaterials. 

Nanomaterials can enter the body through various routes interacting with different cells and biomolecules. For instance, engineered medical nanoparticles used for therapeutic purposes (such as tumor-specific delivery of drugs) are injected into the circulation system so they first interact with blood proteins before being distributed.

Size, shape, ligand density, hydrophobicity, and charge are significant factors when it comes to nano-bio interactions. In order to assess nano-bio interactions, the characterization of nanomaterials is essential. For example, positively charged nanomaterials show an increased rate of absorption by the slightly negatively charged cell membrane, while neutral nanomaterials show the highest blood half-life [3]. Nanomaterials bound with ligands can interact with cells affecting signaling pathways or entering the cell. Because nanoparticles with diameters less than 6 nm may be expelled by the kidneys, they are quickly cleared from the body. However, when the diameter of a nanomaterial is more than 6 nm, it cannot be removed by the kidneys unless it is made up of degradable components like polymers, lipids, or hydrogels [3].

As nanomaterials can have detrimental effects on human health, there has been an uprise of toxicology studies focusing on NMs safety. Nanotoxicology examines the fate of these materials in the human body and their consequences. Various reviews and viewpoints have been reported, along with numerous in situ toxicological research [12,13,14,15,16,17,18]. In order to examine the dangers of NMs in engineering (ENMs) in aquatic systems, several studies, including analytical methodologies and ecotoxicity assessments, have already been published [19,20,21,22,23,24]. An overview of nanomaterials used in food, such as food additives, food packaging materials [25], and cosmetics [26] is provided in some publications and studies. Nanomaterials are mobile, even when they are incorporated in a matrix and their use results in immediate and usually purposeful contact with the human organism [27]. The increased industrial usage of nanoparticles and ultimately in consumer products may lead to higher concentrations of more stable types over time, with possible absorption into the food chain [13]. Measuring standards and formally recognized test methods and recommendations for nanomaterials were developed by the OECD (Organisation for Economic Co-operation and Development) and standardization bodies such as ISO and CEN (European Committee for Standardization) [28].

## 3. Bioanalytical Methods for the Determination of Nanoparticles

Advanced analytical techniques and apparatus have been developed to provide acceptable tools for the characterization of specialized NPs in response to rising demand for their production [29]. The development of analytical methodologies for the determination of nanomaterials according to the European Commission’s Recommendation is vital [30]. 

### 3.1. Electron Microscopy

The use of electron microscopy to characterize NPs in a variety of matrices has a long history. Electron microscopy is regarded as one powerful technique for NP analysis because of its capacity to visualize the NPs and thus obtain information on their size, shape, and state of aggregation. Scanning electron microscopes (SEM) coupled with a field-emission electron gun may match with a spatial resolution of transmission electron microscopy (TEM) of less than 1nm [29].

Conversely, the biggest asset of microscopy resides in the potential of connecting it with other spectroscopic instruments that give information about the structure and the origin of NPs [29]. In the study, the chemical content and morphology of Se NPs were observed in Si-rich yeast, while studied with the use of a TEM equipped with EDX [31]. The sample preparation included mesh copper grinds with holey carbon coatings and a few mL of sample were allowed to dry fully. The method had LOD for the three isotopes of Se (Se78, Se80, Se78) was 7.33, 0.074, and 0.051 μg/L, respectively. The isotope of Se80 was not detected [31]. These data were therefore used for the single-particle analysis, while connected with inductively coupled plasma-mass spectrometry (ICP-MS), in order to accurately find the size of the unknown particles.

### 3.2. Optical Microscopy

Due to the current use of enhanced darkfield hyperspectral imaging (EDF-HSI), this approach was rejuvenated. The use of EDF-HSI with optical spectroscopy allows the brighter appearance of NPs and their full visibility, while their near-infrared spectra can be recorded. The particle illumination intensity is 150-fold due to the high-intensity darkfield conditions. It is important that the EDF-HSI analysis takes place under atmospheric pressure. With this technique, it is possible for liquid samples to be analyzed with little preparation and it can be applied for the research of NP behavior with in-situ, time-dependent monitoring [27]. 

In order to analyze the capabilities of EDF-HIS, Pena et al. conducted a study, which compares the EDF-HIS, energy dispersed X-ray spectroscopy, SEM, and Raman spectroscopy. To serve as a toxicological model for epidermal contamination, porcine skin tissue was subjected to ceria and alumina NPs, and the techniques were employed to determine and identify the NPs. The results show that EDFM-HIS (enhanced dark-field microscopy-hyperspectral imaging) mapping can determine and identify ENMs in tissue, which is supported by traditional approaches. They also provide preliminary verification of EDFM-HSI mapping as a novel and slightly elevated technique for identifying ENMs in biological samples, as well as a foundation for future protocol development using EDFM-HSI for ENM semiquantitative [28].

### 3.3. Light Scattering

High-throughput approaches for analyzing NPs in aqueous media include dispersion methods such as multi-angle light scattering (MALS) and dynamic light scattering (DLS). The average intensities of the dispersed incident light gathered at multiple angles are used to calculate size metrics such as the axis of rotation using MALS. However, DLS uses a time-dependent variation in scattering intensity to estimate the phase transition of the NP. This fluctuation is induced by both beneficial and harmful interferences and is proportional to an identical hydrodynamic diameter [29]. In addition, hyphenation is frequently utilized with size separation devices including hydrodynamic chromatography (HDC) or field flow fractionation (FFF).

Correia and Loeschner recently developed an analytical approach for detecting nano plastics in tissues of fish that uses FFF in conjunction with a MALS detector. The limit of detection of the method was 52 μg/g fish. In this research, another type of nanoplastic in the solution that could be used with the AF4-MALS technique is polyethylene (PE) but the scientists discovered that the background dispersion was too great for the detection of polyethylene NPs [31]. In another study, Deering et. al. used a DLS detector in combination with a sedimentation FFF in order to detect SiO_2_ NPs in lung tissue after enzyme digestions. This research showed that SdFF and tissue digestion add to previous approaches by recognizing unidentified metal oxide nanoparticles and differentiating nanoparticles (with 100 nm diameter) from soluble chemicals and bigger particles with the same nominal elemental composition, thus contributing to the research of natural and artificial nanoparticles [32].

### 3.4. X-ray Tomography

X-ray-based techniques which include X-ray fluorescence (XRF) or transmission X-ray microscopy are other, less widely utilized techniques for imaging NPs (TXM). The former, however, can create two-dimensional images that are accurate representations of the latter technique and can generate three-dimensional pictures by measuring the elemental distribution of the monitored analyte. The spatial resolution of a synchrotron-generated XRF beam is normally between 1 and 10 m, but some XRF beams have a spatial resolution of up to 100 m. Beamlines for sub-micrometer analysis are available and provide a spatial resolution of up to 100 nm [33]. 

In the studies of CeO_2_ NP deposition in rat lung tissue [32], earthworms [33], and zebra fish, the uptake of TiO_2_ and multiwalled nanotubes was estimated [17] with the crucial information from XRF [34]. This technique provided important information for the absorption of Au nanosheets by the skin. 

Imaging techniques cannot distinguish between the analyte’s physical states; a high signal concentration of the analyte does not imply its presence in a particulate form. However, tomography is useful for mapping NP distribution within living creatures in order to understand the molecular mechanisms that contribute to their creation [35,36].

### 3.5. Spectroscopic

ICP coupled with MS is the most frequently used technique for nanoparticle analysis. A variety of methods was developed for different biological matrices [37]. Rat liver was also analyzed by AF4-ICP-MS for the determination of Au NPs by Schmidt et al. [38]. Henss et al. determined Gamma-mercapto-propyl-trimethoxysilane (gamma-MPTS) modified silica-coated magnetic nanoparticles in cultured cells with the use of ETV-ICP-MS and reached a LOD of 0.72 ng/L, 0.86 ng/L, and 1.12 ng/L for Cd, Hg, and Pb respectively [39]. Ag NPs were determined in chicken meat and human tissue by Peters et al. and Vidmar et al. [40,41]. Metal particles and metalloproteins were determined in different biofluids by Loeschner et al. with field-flow fractionation coupled to ICP-MS (AF4-ICP-MS) [42]. Rat liver and kidney were also analyzed by Arslan et al. with sp-ICP-MS for the determination of Cd and Se [43]. As it was made evident by bibliographical research, sp-ICP-MS is the most often utilized technique for metallic NPs. It has been used for the determination of Hg and Se NPs in whale liver and brain by Gajdosechova et al. [44]; Ag NPs and Au NPs in beef, Daphnia manga, and Lumbriculus variegatus; Se NPs in yeast by Gray et al. [45]; platinum nanoparticles (PTNPs) in human urine and blood serum by Fernandez-Trujillo et al. [46]; silver nanoparticles in human tissue by Abdolahpur Monikh et al. [47]; silver and gold nanoparticles in human samples by Bocca et al. [48]; and Au NPs and Ag NPs in human blood by Witzler et al. [49], silver nanoparticles in chicken meat (subjected to in vitro human gastrointestinal digestion, saliva, gastric and intestinal digestions [50]. The LOD for these studies reached the ppt levels. ICP–OES (inductively coupled plasma-optical emission spectrometry) was used for determining Al(III) in hair and scallop reference materials, spiked water samples, and human urine, reaching a LOD of 60 pg/mL [51]. More studies for NPs determination are presented on Table 1 [52,53,54,55,56,57].

## 4. Sample Preparation

Reliable methods for isolating NPs from the tissue without unexpected modifications are needed to examine physical variations of NPs caused by their host environment [43]. The analysis of nanomaterials requires the use of sample preparation techniques. The recent trends in the sample preparation and analysis have already been reviewed by Saleh [58].

Using an acidic or basic environment to degrade tissue might cause particle disintegration or aggregation, giving incorrect changes in the physical condition of the particles. Acid dissolution rate for NP extraction from the biological matrix is typically not recommended because the surface of NPs is affected dramatically. Arslan et al. discovered that sonication of CdSe NPs in 0.5% HCl enhances NP accumulation, most likely as a result of the thiol capping on the particles’ surface dissolving [43]. In addition, the solution had a higher concentration of ionic Cd, indicating that some of the CdSe NPs had disintegrated. However, the influence of alkaline and enzymatic dissolution rate on NPs appears to be more complex, and the published results show that more parameters may influence the efficiency of these approaches. The most common alkali solubilizer is tetramethylammonium hydroxide (TMAH). KOH and NaOH, conversely, were utilized in the process by doctors [59]. While the research that has been published concurs that TMAH is beneficial in tissue solubilization, its influence on NP stability has been questioned. Furthermore, in a 10% (*v*/*v*) TMAH solution, the ultrasonication of CdSe NPs had no effect on the NPs. In addition, the radiant heat of the solution enhanced the NP aggregation and it did not lead to ionic Cd release [29].

The aggregation of AgNPs was detected when biological tissue was dissolved in 20% TMAH, causing the production of AgNPs, when a sample was injected with ionic Ag [7]. In the research of Gray et al., no difference was found in the AgNPs that were recovered from the biological tissue in different TMAH concentrations, however, a minor movement in larger particle size was observed [45]. In addition, for the formation of NPs in biological matrices, enzymatic dissolution is recommended [28]. The most common enzymes that are used in animal tissues are proteases, in conjunction with sodium dodecyl sulfate (SDS) as a stabilizer and sodium azide to inhibit bacterial development [29]. Only incomplete decomposition of muscle tissue was found in the research of Campbell et al. The digestion was carried out at 60 °C resulting in partial decomposition due to protein aggregation [60]. When a combination of hyaluronidase and collagenase was utilized, as well as proteinase K at 65 °C, the recoveries of SiO_2_ were poor [29].

Full degradation of tissue is caused using proteinase K at temperatures ranging from 34 to 37 °C according to research. When proteinase K is utilized, the published data also imply that NPs suffer less, if any, of the physical changes seen under TMAH conditions [29]. Once standard tissue was injected with ionic Ag, no formation or degradation of AgNPs was observed and similarly, no NP production was found.

**Figure 1:**
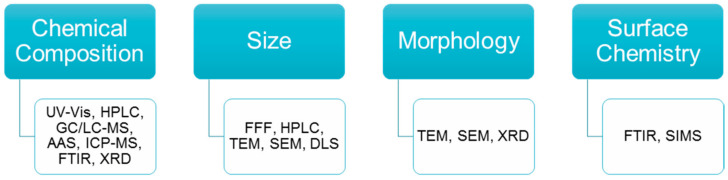
Techniques used for the determination of different parameters of nanomaterials. UV-Vis: Ultraviolet-visible spectroscopy, HPLC: High-performance liquid chromatography, GC/LC-MS: Liquid chromatography/gas chromatography-tandem mass spectrometry, AAS: Atomic absorption spectroscopy, ICP-MS: Inductively coupled plasma mass spectrometry, FTIR: Fourier transform infrared spectroscopy, XRD: X-ray diffraction, FFF: Field-flow fractionation, TEM: Transmission electron microscopy, SEM: Scanning Electron Microscopy, DLS: Dynamic light scattering, SIMS: Secondary-ion mass spectrometry.

Figure 1 summarizes the techniques used for the determination of different parameters of nanomaterials.

## 5. Discussion

Characterization of nanoparticles is a challenging task for analytical chemists. For starters, most current nanoparticle characterization approaches give relatively limited data before or after designed nanoparticles enter the biological milieu. New strategies for extracting and preconcentrating nanoparticles from biological matrices before physic-chemical characterization are likely to be developed in the next years. The techniques frequently utilized for this purpose are electron microscopy, scanning probe microscopy, and dynamic light scattering, which disclose nanoparticle size and dispersion but not aggregation/agglomeration, biomolecule adsorption, or biotransformation. An image of the surface of a sample can be obtained by SEM or TEM microscopy offering data about the physical characteristics of the nanomaterials. These techniques though are destructive to the sample and time-consuming [40]. Spatial resolution for SEM ranges between 5 and 100 nm and for TEM can be less than 1 nm. Higher spatial resolution means bigger portions of the sample can be analyzed and more reliable data can be obtained. NMs can change in size in a biological matrix, increase due to aggregation, or decrease due to ion release for example. The wider the size range the more impractical it becomes to measure size distribution reliably [55,56].

Another technique that is commonly used for size analysis of NMs, especially for uniformly dispersed samples, is dynamic light scattering (DLS). One drawback of DLS is that small particles are not detected in complex matrices containing large particles and aggregates. Qualitative analysis with minimal sample preparation can be conducted with X-Ray absorption spectroscopy. Information about the composition of NMs can be obtained with FT-IR. The elemental makeup of a homogeneous material can be determined accurately using LIBS. There are no defined protocols, to the best of our knowledge, for size characterization because of the wide range of NMs and analytical approaches.

UV/Vis techniques, fluorescence techniques, NIR-fluorescence, infrared spectroscopy, and Raman spectroscopy are all examples of spectroscopic techniques. Fluorescence spectroscopy is effective for assessing both the quantitative and qualitative aspects of nanoparticle uptake and localization. Although no label permits natural samples to be detected, non-natively fluorescent nanostructures can be analyzed using fluorescence methods after using one of many available labeling approaches to study uptake in toxicological experiments.

For nanoparticle characterization, optical-spectral approaches are both informative and practicable. These methods have several advantages, including good sample representativity, simple sample preparation, increased efficiency of analysis and the potential for automation, and low cost of measuring instrumentation. The majority of optical spectrum techniques also allow for direct measurements of nanoparticle parameters in liquids. On many occasions, it is required to combine the information of optical-spectral approaches with the results from high-resolution microscopy.

The determination of a nanoparticle’s size includes scanning and transmission electron microscopy, dynamic light scattering, and size-exclusion chromatography. The information about the size values acquired by these methods can differ. Size separation and extraction of nanoparticles in liquids can be accomplished via size-exclusion chromatography, ultrafiltration, and field-flow fractionation (FFF) [34]. The last technique can be a perfect choice for immediate separation, detection, and characterization of nanoparticles since it enables the separation in a liquid biological matrix, and it gives a size distribution as a high-resolution method. Carbon nanotubes, particles, cells, bacteria, viruses, and natural organic materials have all been separated using FFF. This approach was used to characterize, isolate, and quantify unlabeled inorganic nanoparticles collected from a biological medium. The necessity for standards to calibrate the FFF apparatus is arguably the most significant shortcoming of this technology. The most useful approximation to data is the usage of polymeric nanoparticles.

Sub-ppb detection limits, excellent precision, and a dynamic range of five orders of magnitude or more are advantages of ICP-AES. The cellular and nanoparticle sources of carbon are difficult to differentiate through this technique, since it is limited to metallic nanoparticles, confined elements, and dissolved ions that have been released from the nanomaterial. Thus, the position of the nanoparticles cannot be determined. ICP-MS can also be used to evaluate material using comparable processes. Mass spectrometry is a more sensitive detector than optical emission spectroscopy (OES). MS detectors also have the ability to examine isotopes and perform a multi-analysis in a single run.

The most common approach is that of ICP-MS in single-particle mode (sp-ICP-MS) [42,52,53]. This technique provides information about the elemental composition and concentration of NMs in suspensions. The preparation of samples for sp-ICP-MS is vital. Individual monitoring of nanoparticles per dwell period requires optimal dilution. The elimination of interfering matrices is critical in order to reduce background noise and variations in transport efficiency. This method works well with ENP samples but badly with real samples. A compelling solution for overcoming this problem is the connection of sp-ICP-MS with a fractionation/separation, in order to overcome these challenges and achieve the best results.

## 6. Conclusions

This review summarized the analytical techniques used for the determination of nanomaterials in biological matrices. Precision techniques are able to provide detailed molecular information, and the tailoring of these techniques could be achieved by taking into consideration the needs of sample preparation. Ultimately, the development of analytical methodologies that could monitor in situ and in real-time nanoparticles in a wide range of biomedical applications is essential.

## Figures and Tables

**Table 1:** List of analytical methods used for nanomaterial determination/characterization in biological matrices.

Analyte	Matrix	Analytical Method	LOD	Ref
metal particles and metalloproteins	biofluids	AF4-ICP-MS		[42]
Au NPs	Rat liver	AF4-ICP-MS		[48]
Cd, Pb, Hg	Cultured cells	ETV-ICP-MS	Cd (0.72 ng/L), Hg (0.86 ng/L), Pb (1.12 ng/L)	[39]
Ag NPs	Chicken meat	sp-ICP-MS		[40]
Ag NPs	Human tissue	sp-ICP-MS		[41]
CdSe	Rat liver and kidney	sp-ICP-MS		[43]
HgSe NPs	Whale liver and brain	sp-ICP-MS		[44]
Ag NPs, Au NPs	Beef, Daphnia magna, Lumbriculus variegatus	sp-ICP-MS		[45]
Platinum nanoparticles PTNPs	human urine, blood serum	SP-ICP-MS	1.9 × 10^5^ particles/L	[47]
Silver nanoparticles	human tissue	(SP-ICP-MS)		[46]
Silver and gold nanoparticles	human samples	SP-ICP-MS, AF4-FFF-MALS-UV-ICP-MS	0.0006 ng/mL	[48]
silver nanoparticles	chicken meat subjected to in vitro human	SP-ICP-MS	0.5 ng/L	[50]
	gastrointestinal digestion			
	saliva, gastric and intestinal digestions.			
Au NPs	human blood	SP-ICP-MS	Ag 15 ng/L	[49]
Ag NPs			Au 25 ng/L	
Al(III)	Hair and scallop reference	ICP-OES	60 pg/mL	[51]
	materials, spiked water samples,			
	human urine			
Cr(III), Cr(VI)	Drinking water	ICP-MS	Cr(III) 1.5 ng/L	[52]
			Cr(VI) 2.1 ng/L	
Cd(II)	Water, wastewater, biological	FAAS	0.14 lg/L	[53]
	and food samples			
Pb(II), Cr(III)	Various water, food, industrial	FAAS	Pb(II) 0.43 lg/L	[54]
	effluent, and urine samples		Cr(III) 0.55 lg/L	
As(III), As(V), Sb(III), Sb(V)	natural waters	ICP-OES	on-line: As(III) 0.53 ng/mL,	[55]
			As(V) 0.49 ng/mL	
			Sb(III)0.77 ng/mL,	
			Sb(V) 0.71 ng/mL	
			off-line: As(III) 0.11 ng/mL	
			As(III) 0.11 ng/mL,	
			As(V) 0.10 ng/mL	
			Sb(III)0.15 ng/mL,	
			Sb(V) 0.13 ng/mL	
Mn, Cd	Water samples	FAAS	Mn (1.0 ng/mL),	[56]
			Cd (0.96 ng/mL)	
Cd, Cr, Cu, Mn	Environmental samples	ICP-OES	Cd (48 ng/L), Cr	[57]
			Cr 36 ng/L	
			Cu 21 ng/L	
			Mn 24 ng/L	

Ref.–Reference.

## Data Availability

Not applicable.
